# Significance of mesothelin expression in preoperative endoscopic biopsy specimens for colorectal cancer prognosis

**DOI:** 10.18632/oncotarget.27774

**Published:** 2020-10-27

**Authors:** Takehiro Shiraishi, Eiji Shinto, Masato Yamadera, Ken Nagata, Hitoshi Tsuda, Satsuki Mochizuki, Yoshiki Kajiwara, Koichi Okamoto, Takahiro Einama, Yoji Kishi, Hideki Ueno

**Affiliations:** ^1^Department of Surgery, National Defense Medical College, Tokorozawa, Saitama 359-0042, Japan; ^2^Department of Basic Pathology, National Defense Medical College, Tokorozawa, Saitama 359-0042, Japan

**Keywords:** colorectal cancer, mesothelin, endoscopic biopsy specimen, immunohistochemistry, Pathology

## Abstract

Mesothelin (MSLN) is a cell surface glycoprotein that is normally expressed in the mesothelial cells but highly expressed in several malignant tumors, where the high expression is generally associated with poor prognosis. In this work, 512 patients with stage III colorectal cancer (CRC) were examined to ascertain the prognostic value of MSLN expression in preoperative endoscopic biopsy specimens. MSLN expression was evaluated by immunohistochemical staining. The tumor cells were MSLN-positive in 61 of the 512 patients (11.9%). MSLN expression was associated with a shorter disease-specific survival (DSS) period (5-year DSS = 68.7%, *P* = 0.0008). Besides, by multivariate analysis, MSLN expression was identified to be a marker of poor prognosis by multivariate analysis (*P* = 0.0033, hazard ratio (HR) = 2.31) as well as macroscopic type (*P* = 0.047, HR = 1.82) among the factors that can be evaluated preoperatively. MSLN-positive patients had a significantly poorer prognosis regardless of adjuvant chemotherapy administration (*P* = 0.0081 and *P* = 0.0018 for surgery alone and chemotherapy, respectively). MSLN-positive patients in the adjuvant chemotherapy group exhibited a significantly lower risk of recurrence when compared with those in the surgery alone group (*P* = 0.0090). In conclusion, high MSLN expression observed in preoperative endoscopic biopsy specimens of stage III CRC was an independent poor prognostic factor. Preoperative evaluation of MSLN by immunohistochemical staining might be applied to select individuals for intensive preoperative chemotherapy among the stage III CRC patients.

## INTRODUCTION

Colorectal cancer (CRC) is one of the most common cancers worldwide and is the second most frequent cause of cancer-related mortality in Japan [1]. For stage II/III CRC, the standard treatment in the country is to initially perform radical resection and subsequently provide adjuvant chemotherapy based on the pathological findings of the resected specimen [2]. Since the effect of chemotherapy after the formation of a cancerous mass is generally limited [3], early chemotherapy is preferred. Recently, short-term results were reported from a national trial (FOxTROT trial) in which oxaliplatin-based neoadjuvant chemotherapy was administered for CRC with predicted stages T3-4, N0-2, and M0. Patients in the neoadjuvant chemotherapy group tended to have lower 2-year postoperative recurrence rates (neoadjuvant chemotherapy *vs*. postoperative adjuvant chemotherapy, 13.6% *vs*. 17.2%; *P* = 0.08) [4]. If patients with a high risk of recurrence could be identified prior to treatment, the benefit of preoperative treatment could be evaluated in this group.

Changes in the invasive frontal margin of CRC, such as an activation of the Wnt signaling pathway, are considered to reflect malignancy. Several genes have already been identified, the expression of which correlates with prognosis [5–7]. However, we continue to hope that molecular tests that make use of the surface tissue will help in deciding tailor-made treatment strategies, including preoperative therapy based on cancer biological characters. This is because endoscopic biopsy from the cancer surface remains to be the only way to obtain cancer tissue before treatment.

Mesothelin (MSLN) is a 40-kDa cell surface glycoprotein, and its expression in normal human tissue is limited to the mesothelial cells lining the pleura, pericardium, and peritoneum [8, 9]. Moreover, MSLN is highly expressed in several malignant tumors, and its expression, which is reportedly related to the Wnt signaling pathway [10], might be associated with patient prognosis [11, 12]. For stage II/III CRC, we previously demonstrated that MSLN expression is a robust independent prognostic factor using standard sections, which were the maximum sections that included the invasive margin of the cancer [13]. In addition, we evaluated the expression of MSLN in four specific areas (i.e., submucosa, subserosa, central area, and superficial tumor area) by immunohistochemical staining and found that MSLN expression exhibits little heterogeneity and correlates with poorer prognosis regardless of the area [14].

This study aimed to understand the association between MSLN expression in preoperative endoscopic biopsy specimens and unfavorable prognosis in stage III CRC. Our previous observations revealed that approximately 50% of patients with stage III CRC who show high MSLN expression develop postoperative recurrence [13]. We envisaged that accurate characterization of this type of cancer in stage III CRC prior to the treatment could aid in the selection of patients who would benefit from a more intensive therapeutic approach. Furthermore, the homogeneity of MSLN expression was reconfirmed by comparing the expression in the biopsy tissue with that in the invasive frontal margin of the cancer.

## RESULTS

### Interobserver agreement and correlations between immunohistochemical analysis of MSLN and clinicopathological characteristics

**Figure 1:**
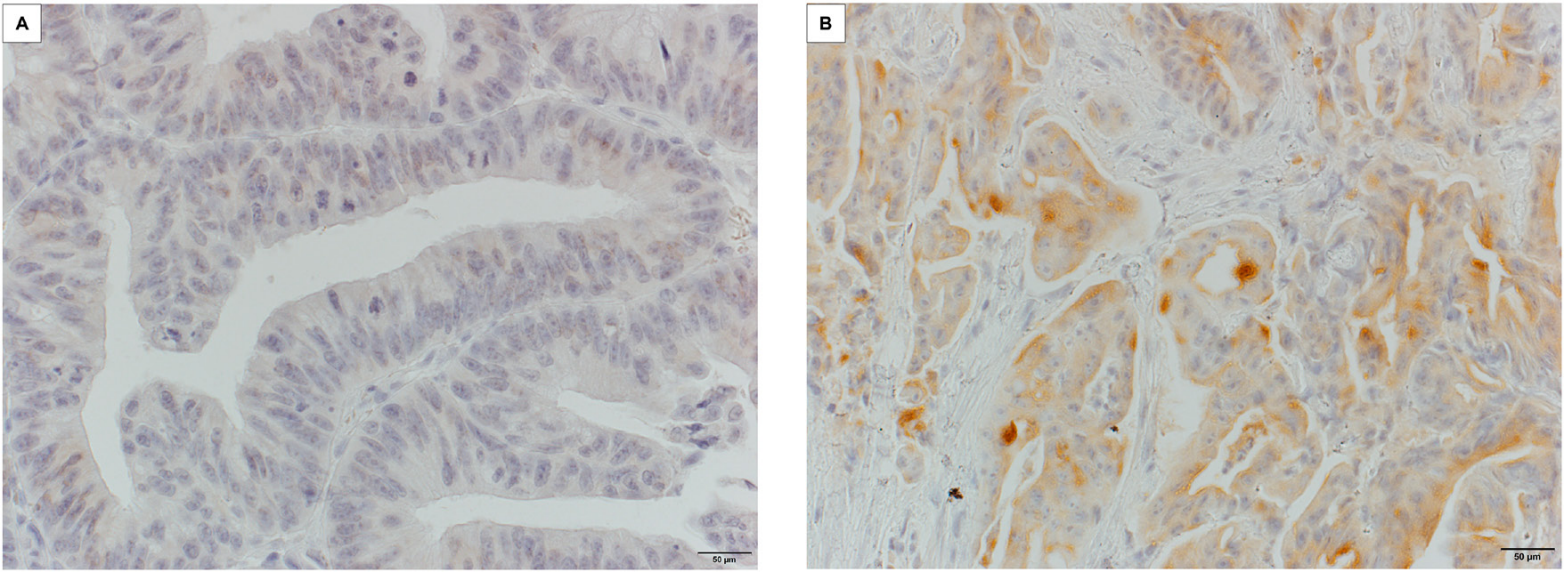
Microscopic appearance of MSLN expression in CRC tissue. (**A**) MSLN-negative tissue with no stained cells. (**B**) Positive staining at the endoluminal surface or in cytoplasmic deposits/granules. (Magnification: A, B 400×). MSLN, mesothelin; CRC, colorectal cancer.

Microscopic appearance of MSLN expression is shown in Figure 1 and the clinicopathological characteristics of the study participants are summarized in Table 1. Of the 512 patients, 61 (11.9%) were found to be MSLN-positive. Interobserver agreement with regard to the evaluation of MSLN immunostaining was substantial (85.7%, κ = 0.64). Table 1 shows the correlation between MSLN immunoreactivity and clinicopathological characteristics. The expression did not correlate with any of the clinicopathological characteristics. The concordance rate between biopsy specimens and the frontal margin of the tumor with regard to MSLN expression was 87.4% (*P* < 0.0001) (Supplementary Table 1).

**Table 1:** Correlation between clinicopathological characteristics and mesothelin expression

Parameters	Categories	Total (*n* = 512) *n*(%)	Mesothelin expression		
			Negative (*n* = 451) *n* (%)	Positive (*n* = 61) *n* (%)	*P* value
Sex	Male	289 (56.4)	260 (57.7)	29 (47.5)	0.14
	Female	223 (43.6)	191 (42.3)	32 (52.5)	
Age (years)	< 65	230 (44.9)	196 (43.5)	34 (55.7)	0.071
	≥ 65	282 (55.1)	255 (56.5)	27 (44.3)	
Location	Right side	112 (21.9)	103 (22.8)	9 (14.8)	0.14
	Left side	400 (78.1)	348 (77.2)	52 (85.2)	
Serum CEA level before surgery (μg/L)	≤ 5.3	334 (65.2)	291 (64.5)	43 (70.5)	0.35
	> 5.3	178 (34.8)	160 (35.5)	18 (29.5)	
Serum CA19-9 level before surgery (U/ml)	≤ 37	421 (82.2)	375 (83.2)	46 (75.4)	0.15
	> 37	91 (17.8)	76 (16.8)	15 (24.6)	
Macroscopic type^*1, 5^	Type 0/1/2	461 (90.0)	410 (90.9)	51 (83.6)	0.095
	Type 3/4/5	51 (10.0)	41 (9.1)	10 (16.4)	
Maximum diameter of tumor (mm)^*5^	< 50	275 (53.7)	240 (53.2)	35 (57.4)	0.54
	≥ 50	237 (46.3)	211 (46.8)	26 (42.6)	
Tumor intestinal annular propotion^*5^	< 50	136 (26.6)	117 (25.9)	19 (31.1)	0.39
	≥ 50	376 (73.4)	334 (74.1)	42 (68.9)	
Histopathological grading^*2^	G1	170 (33.2)	154 (34.1)	16 (26.2)	0.39
	G2	298 (58.2)	260 (57.7)	38 (62.3)	
	G3	44 (8.6)	37 (8.2)	7 (11.5)	
Depth of tumor^*2, 5^	≤ T2	61 (11.9)	55 (12.2)	6 (9.8)	0.58
	≥ T3	451 (88.1)	396 (87.8)	55 (90.2)	
Lymph node metastasis^*2^	N1	364 (71.1)	324 (71.8)	40 (65.6)	0.32
	N2	148 (28.9)	127 (28.2)	21 (34.4)	
Pathological stage^*2^	Stage ΙΙIA	54 (10.6)	48 (10.6)	6 (9.8)	0.86
	Stage ΙΙΙB	354 (69.1)	313 (69.4)	41 (67.2)	
	Stage ΙΙΙC	104 (20.3)	90 (20.0)	14 (23.0)	
Venous invasion	Absence	34 (6.6)	30 (6.7)	4 (6.6)	0.98 ^*4^
	Presence	478 (93.4)	421 (93.3)	57 (93.4)	
Lymphatic invasion	Absence	11 (2.1)	10 (2.2)	1 (1.6)	0.77 ^*4^
	Presence	501 (97.9)	441 (97.8)	60 (98.4)	
Tumor budding^*1^	BD1, 2	315 (61.5)	284 (63.0)	31 (50.8)	0.070
	BD3	197 (38.5)	167 (37.0)	30 (49.2)	
Mismatch repair protein^*3^	Proficient	476 (93.0)	418 (92.7)	58 (95.1)	0.49 ^*4^
	Deficient	36 (7.0)	33 (7.3)	3 (4.9)	
Adjuvant chemotherapy	Surgery alone	172 (33.6)	158 (35.0)	14 (23.0)	0.054
	Chemotherapy	340 (66.4)	293 (65.0)	47 (77.0)	

^*^1 Japanese Classification of Colorectal Carcinoma (9th Edition, 2018); ^*^2 TNM Classification (8th Edition, 2017); ^*^3 Mismatch repair protein status was verified using immunohistochemical staining of MLH1 and MSH2; ^*^4 Fisher’s exact test; ^*^5 Based on preoperative examinations.

### Correlation between MSLN immunohistochemistry and mRNA expression

**Figure 2:**
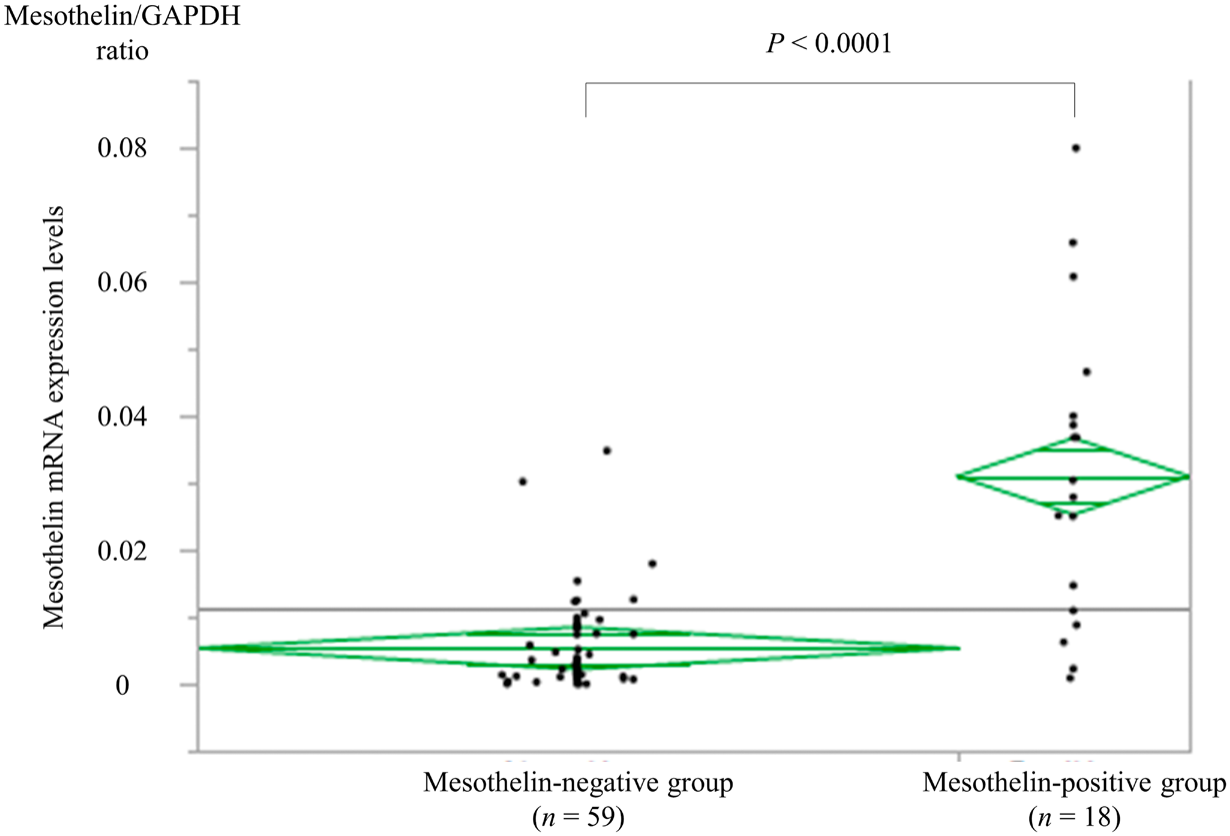
Correlation between immunohistochemistry and mRNA expression. Cancer tissue samples found to be MSLN-positive (*n* = 18) by immunohistochemistry had significantly higher MSLN mRNA expression (median MSLN/GAPDH ratio, 0.029; range 0.00088–0.080) than the MSLN-negative tissues (*n* = 59) (median MSLN/GAPDH ratio, 0.00324; range 0.0000011–0.035, *P* < 0.0001). MSLN, mesothelin; GAPDH, glyceraldehyde 3-phosphate dehydrogenase.

Of the 77 evaluated cancers, 18 were MSLN-positive (23.4%) and 59 were negative (76.6%). Cancers exhibiting MSLN-positive immunostaining had significantly higher MSLN mRNA expression (median MSLN/GAPDH ratio, 0.031; range, 0.025–0.037) than the MSLN-negative ones (median MSLN/GAPDH ratio, 0.0053; range, 0.0022–0.0085; *P* < 0.0001; Figure 2).

### Prognostic implications of MSLN status

**Figure 3:**
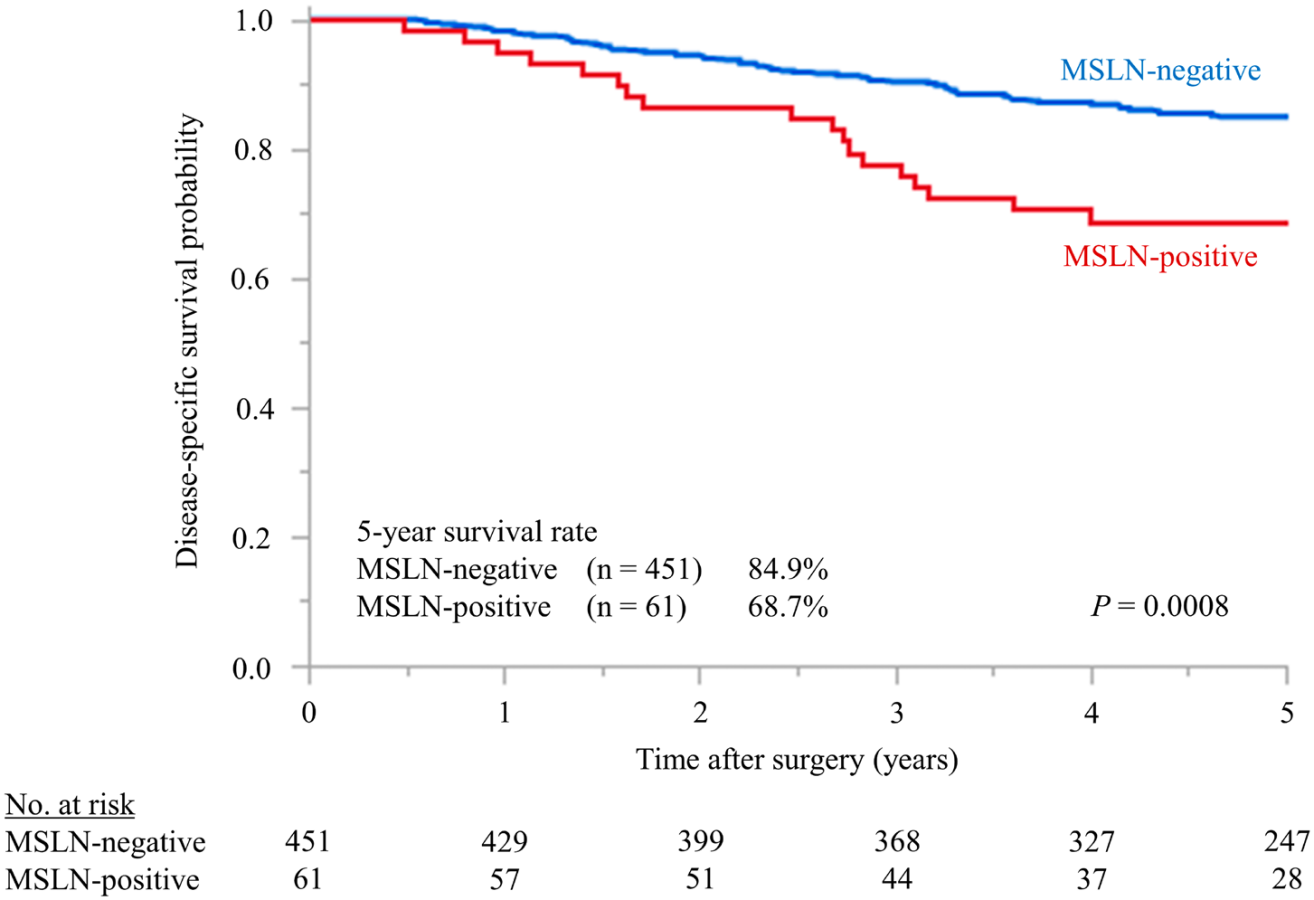
Kaplan–Meier survival estimates of stage III CRC patients with differences in MSLN expression detected by immunohistochemistry. MSLN-negative *vs.* MSLN-positive patients with stage III CRC. The difference in 5-year DSS (84.9% *vs.* 68.7%) was significant (*P* = 0.0008). MSLN, mesothelin; DSS, disease-specific survival; CRC, colorectal cancer.

The 5-year DSS rates differed significantly between the stage III CRC patients with MSLN-positive (68.7%) and MSLN-negative (84.9%) tumors (*P* = 0.0008, Figure 3). When applying indices that could be evaluated preoperatively, univariate analysis using the Cox proportional hazards model revealed that the presurgical serum CEA level (*P* = 0.017), presurgical serum CA19-9 level (*P* = 0.049), macroscopic type (*P* = 0.0076), tumor intestinal annular proportion (*P* = 0.0049), histological grading (*P* = 0.034), tumor depth (*P* = 0.0013), and MSLN expression (*P* = 0.0028) correlated significantly with the risk of death from CRC recurrence. Following the Cox multivariate proportional hazard model analysis, upon including variables with *P* < 0.1, MSLN expression remained independently associated with poor DSS in stage III CRC (*P* = 0.0033; HR = 2.31) as well as macroscopic type (*P* = 0.047; HR = 1.82) (Table 2).

**Table 2:** Univariate and multivariate analyses based on Cox’s Proportional Hazards Model for disease-specific survival according to the clinicopathological features evaluated before surgery in stage III colorectal cancer patients

Variables	No. of cases	Univariate Analysis			Multivariate Analysis		
		Hazard Ratio	95% Confidence Interval	*P* value	Hazard Ratio	95% Confidence Interval	*P* value
Sex							
Male/Female	289/223	1.02	0.67-1.55	0.93			
Age							
≥ 65/< 65	282/230	1.36	0.89–2.09	0.15			
Location							
Left side/Right side	400/112	1.26	0.76–2.21	0.38			
Serum CEA level before surgery (μg/L)							
> 5.3/≤ 5.3	178/334	1.67	1.10–2.52	0.017		Not selected	
Serum CA19-9 level before surgery (U/ml)							
> 37/≤ 37	91/421	1.66	1.00–2.63	0.049		Not selected	
Macroscopic type							
Type3, 4, 5/Type0, 1, 2	51/461	2.24	1.26–3.73	0.0076	1.82	1.00–3.09	0.047
Maximum diameter of tumor (mm)							
≥ 50/< 50	27/275	1.46	0.97–2.22	0.071		Not selected	
Tumor intestinal annular propotion							
≥ 50/< 50	376/136	2.16	1.25–4.08	0.0049		Not selected	
Histological grading							
G3/G1, 2	44/468	2.00	1.06–3.48	0.034		Not selected	
Depth of tumor							
≥ T3/≤ T2	451/61	4.37	1.64–17.79	0.0013		Not selected	
Mismatch repair protein							
Deficient/Proficient	36/476	1.06	0.45-2.14	0.88			
Mesothelin expression							
Positive/Negative	61/451	2.32	1.36–3.78	0.0028	2.31	1.34–3.79	0.0033

### Subgroup analyses

**Figure 4:**
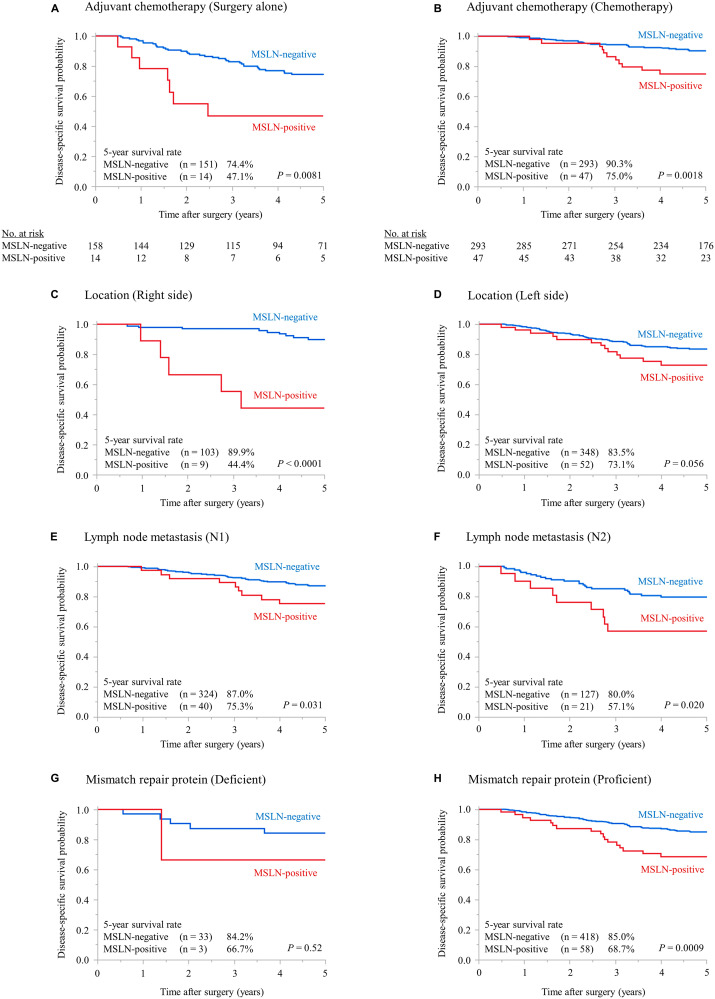
Subgroup analyses. Kaplan–Meier plots for DSS according to the MSLN status in patients who did not receive adjuvant chemotherapy (**A**) or received adjuvant chemotherapy (**B**), whose tumor location was right-sided (**C**) or left-sided (**D**), whose lymph node metastatic status was N1 (**E**) or N2 (**F**), and who were proficient (**G**) or deficient (**H**) in mismatch repair protein. MSLN, mesothelin; DSS, disease-specific survival.

Subgroup analyses are depicted in Figure 4. Figure 4A and 4B compare the DSS between MSLN-positive and negative patients according to the administration of adjuvant chemotherapy (surgery alone or chemotherapy). MSLN-positive patients had a significantly poorer prognosis regardless of the chemotherapy (*P* = 0.0081 and *P* = 0.0018 for surgery alone and chemotherapy, respectively).

Figure 4C and 4D compare the DSS according to tumor location. Right-sided MSLN-positive colon cancer patients had a significantly poorer prognosis (*P* < 0.0001); likewise, among the left-sided colon cancer patients, the MSLN-positive group tended to exhibit a poorer prognosis (*P* = 0.056).

Figure 4E and 4F compare the DSS according to lymph node metastasis status (N1, N2). The MSLN-positive group had a significantly poorer prognosis regardless of the N-stage (*P* = 0.031 and *P* = 0.020 for N1 and N2, respectively).

Figure 4G and 4H compare the DSS according to MMR protein status. Within the MMR-proficient group, MSLN-positive patients displayed a significantly poorer prognosis (*P* = 0.0009). Conversely, among the MMR-deficient patients, no differences in DSS were observed (*P* = 0.52).

### Efficacy of adjuvant chemotherapy based on MSLN expression

**Figure 5:**
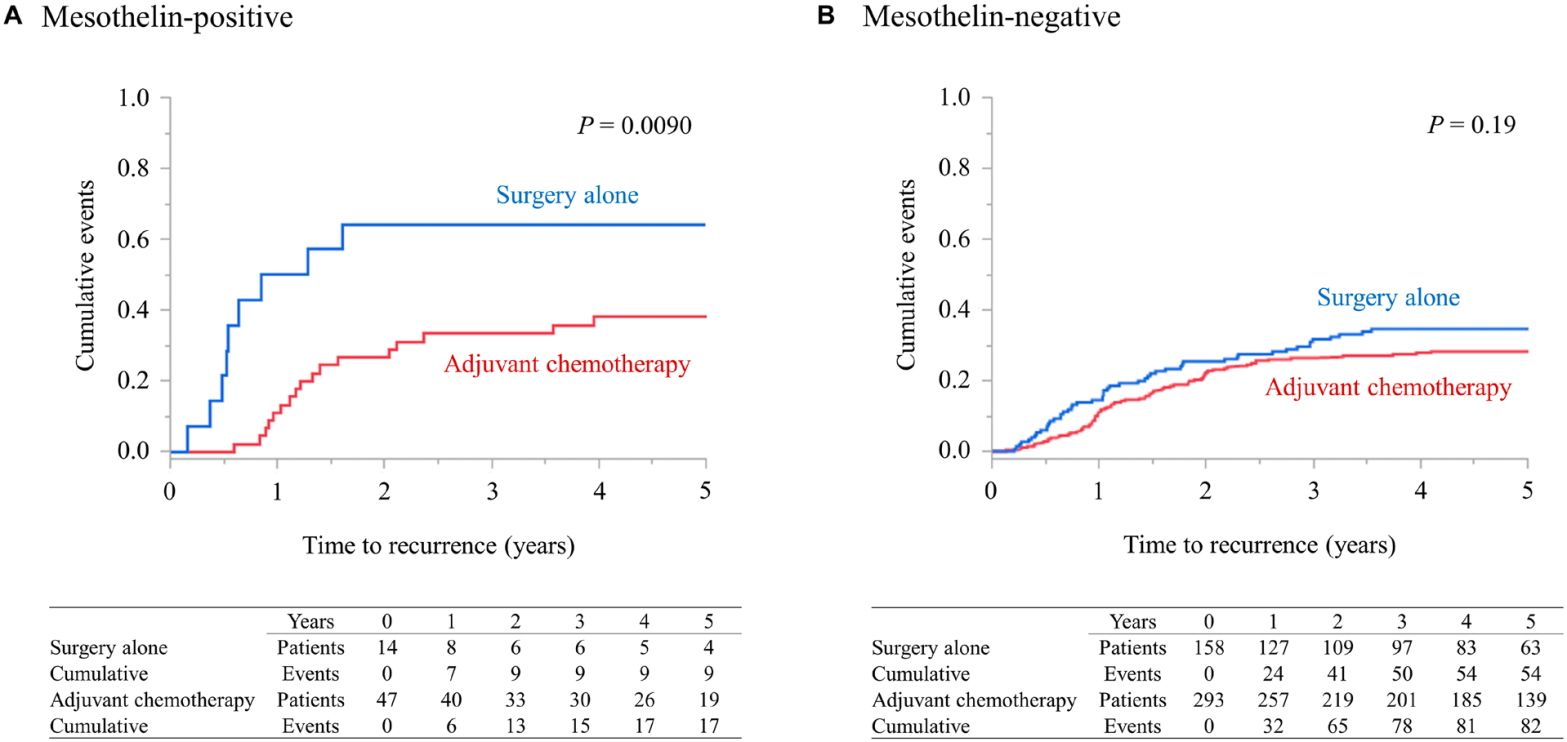
Efficacy of adjuvant chemotherapy according to MSLN expression. (**A** and **B**) depict comparisons of TTR between patients who received surgery alone and those who received adjuvant chemotherapy according to their MSLN status. MSLN, mesothelin; TTR, time to recurrence.

Figure 5A and 5B compare the TTR data between the ‘surgery alone’ and ‘adjuvant chemotherapy’ groups according to MSLN status. MSLN-positive patients in the adjuvant chemotherapy group exhibited a significantly lower risk of recurrence than those in the surgery alone group (*P* = 0.0090). Conversely, within the MSLN-negative group, no differences in TTR were observed (*P* = 0.19).

### Postrecurrent disease-specific survival rate

**Figure 6:**
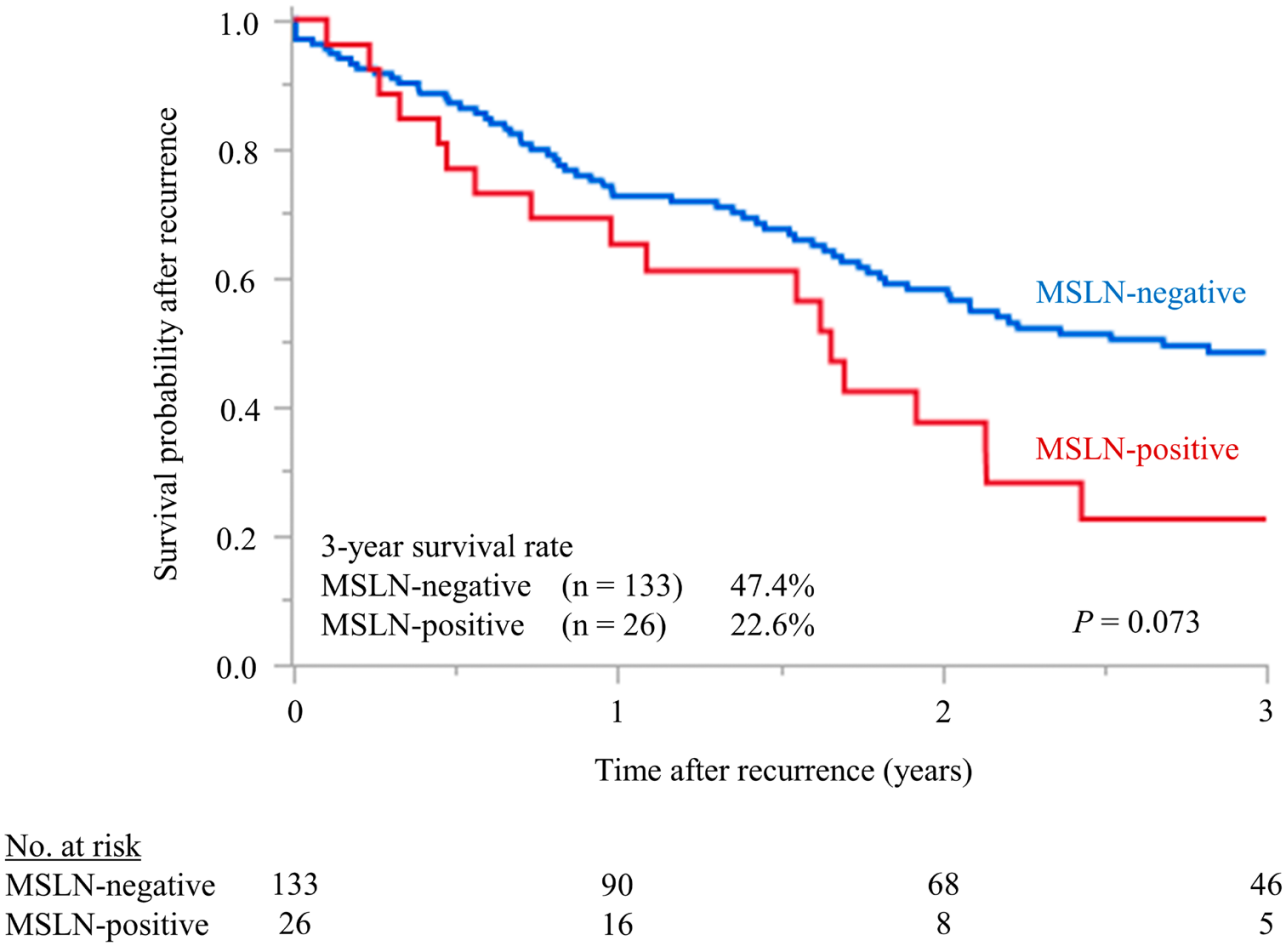
Postrecurrent disease-specific survival rate. Compares the survival probabilities after recurrence between the MSLN-positive and negative patients. After recurrence, MSLN-positive patients tended to have a poorer survival; that is, the 3-year survival rates were 22.6% in the MSLN-positive group and 47.4% in the MSLN-negative group (*P* = 0.073).

Figure 6 compares the survival probabilities after recurrence between the MSLN-positive and negative patients. After recurrence, MSLN-positive patients tended to have a poorer survival; that is, the 3-year survival rates were 22.6% in the MSLN-positive group and 47.4% in the MSLN-negative group (*P* = 0.073).

## DISCUSSION

We previously reported that immunohistochemically-determined MSLN expression is a strong independent prognostic factor in stage II/III CRC when standard sections are used and that the MSLN expression detected in tumors exhibited only limited heterogeneity [13, 14]. In this study, immunohistochemical staining was performed, and the correlation between MSLN expression in preoperative endoscopic biopsy specimens and patient prognosis was examined. We found that the expression was associated with a poor prognosis but not in MMR-deficient patients. The concordance rate of MSLN expression between the biopsy specimens and the frontal margin of the tumor was 87.4% (*P* < 0.0001), verifying the previously identified degree of homogeneity in MSLN expression.

In this study, MSLN-positive patients receiving surgery alone had a markedly high recurrence rate (64%), whereas those receiving adjuvant chemotherapy exhibited a recurrence rate of merely 36%. In patients with recurring MSLN-positive cancer, the 3-year survival rate after recurrence was 22%. These data indicate that adjuvant chemotherapy for remnant small cancer foci might be effective in spite of MSLN positivity. However, MSLN-positive cancer recurrence typically has serious implications on prognosis. Regarding epithelial ovarian carcinoma, high levels of MSLN expression have been frequently detected in chemotherapy-resistant gross masses, which is consistent with our results [15]. In locally advanced CRC cases without metastatic disease and identified as MSLN-positive by endoscopic biopsy, early intensive chemotherapy for eradicating micrometastatic foci might be the most appropriate treatment strategy. Although the correlation between MSLN expression and chemotherapy effect is still unclear, MSLN is expected to serve as a promising marker for selecting patients for neoadjuvant chemotherapy (NAC), a new hopeful therapeutic strategy.

Meanwhile, MSLN-negative cases showed relatively favorable 5-year DSS rates (90%) in the adjuvant chemotherapy group. Given that fluoropyrimidine-based regimens were used in majority (94%) of the patients, it is conceivable that MSLN-negative group could include several patients not requiring oxaliplatin-based regimens. As the high incidence of adverse events related to oxaliplatin therapy is now regarded as a clinical problem, there is a pressing need to develop an algorithm for selecting patients not requiring oxaliplatin. We suggest that MSLN negativity could be a candidate element in this algorithm.

Epithelial mesenchymal transition (EMT) is closely associated with tumor infiltration [16]. Reports have indicated that MSLN may be involved in tumor progression and metastasis through EMT activation [17]. However, there is little difference in MSLN expression between the leading edge of the tumor and the tumor surface. Thus, it may be possible that MSLN is not a molecule that appears along with EMT promotion although it may induce EMT. Argani *et al*. studied 60 cases of pancreatic cancer. MSLN expression was observed in all cancer tissues by immunohistochemistry, while its absence was consistently noted in non-cancerous pancreatic ductal epithelium in patients with and without pancreatitis [11]. This result suggests that MSLN plays a role in tumorigenesis. Assuming that MSLN is related to the process of carcinogenesis and induces EMT, it is plausible that biopsy specimens taken from the cancer surface can be used for the preoperative detection of CRC with high malignancy potential.

The limitations of the present study include potential changes in tissue MSLN antigenicity associated with the processing steps, right from fixation to section preparation. These changes could lower the sensitivity of detection. However, the evaluation of MSLN expression by immunohistochemical staining was validated by RT-qPCR, showing that the procedures were reliable. Second, the retrospective study design, postoperative adjuvant chemotherapy, and surgical procedures that were influenced by age or performance status might have resulted in bias. Third, pathological stage III cannot be diagnosed preoperatively, which means that this study could not reveal the significance of MSLN expression in clinical stage III CRC patients. However, clinical stage III may include various status of cancer; thus, a retrospective study on pathological stage III cancer patients is a necessary first step.

In conclusion, in the present study, high MSLN expression observed in preoperative endoscopic biopsy specimens of stage III CRC was an independent poor prognostic factor. Preoperative evaluation of MSLN by immunohistochemical staining might be useful in predicting the recurrence risk. Further investigations are necessary to understand the usefulness of MSLN in selecting stage III CRC patients for NAC.

## MATERIALS AND METHODS

### Patients

We enrolled 512 patients diagnosed with stage III CRC who underwent curative surgical resection between January 1999 and December 2012 at the National Defense Medical College Hospital, Saitama, Japan. Their medical records were reviewed. Patients with curatively resected and histologically proven stage III (any T, N1-2, M0) CRC were included in the study [18]. Patients with multiple CRCs and multiple primary cancers and those who had received preoperative chemotherapy or radiotherapy were excluded. Macroscopic type and tumor intestinal annular proportion were endoscopically evaluated. Maximum tumor diameter was estimated using X-ray images taken during preoperative barium enema, and the depth of the tumor was evaluated by referring to the endoscopic and barium enema findings. Blood vessel and lymphatic invasions were recorded as being absent or present, and tumor budding was evaluated as per the Japanese Classification of Colorectal, Appendiceal, and Anal Carcinoma: The 3rd English Edition [19].

The mean postoperative follow-up period was 60.3 months, during which 159 patients (31.1%) experienced recurrence. Of the 512 stage III CRC patients, 340 received adjuvant chemotherapy. Among them, 320 received the therapy according to fluoropyrimidine-based regimens (oral tegafur-uracil/leucovorin [UFT/LV], *n* = 278; oral tegafur-uracil [UFT], *n* = 10; 5-fluorouracil/leucovorin [5-FU/LV], 5; capecitabine, *n* = 14; tegafur/gimeracil/oteracil [S-1], *n* = 13) and 20 received it as per oxaliplatin-based regimens (FOLFOX, *n* = 7; CAPOX, *n* = 13). This study was approved by the Institutional Review Board of the National Defense Medical College, Saitama, Japan. Written informed consent for the experimental use of tissue samples was obtained from each patient as per the institutional regulations.

### Immunohistochemical staining and evaluation

Immunohistochemical staining was performed as described previously [20]. Endoscopic biopsy specimens and surgically resected specimens were immunostained for MSLN (clone 5B2 diluted 1:30; Novocastra, Newcastle Upon Tyne, UK). The anti-MSLN antibody used in the study was raised against a recombinant protein corresponding to the membrane-bound form of the MSLN molecule [21].

The slides of immunostained biopsy specimens were evaluated independently by two observers (TS, ES) who were both unaware of the clinical outcomes. The extent of MSLN immunostaining of the tumor cells was evaluated. Biopsy samples subjected to MSLN staining were scored according to the proportion of immune-positive cells as either positive (≥ 10%) or negative (< 10%) (Figure 1). The cutoff score for positivity (10% positive cells) was based on receiver operating characteristic (ROC) curve analysis of death from CRC recurrence within five postoperative years. MSLN-positive staining included positive staining of the apical/endoluminal surface and positive staining of the cell membrane and/or cytoplasm. If the results were not in agreement between the observers, a consensus was reached after reevaluation. The degree of interobserver agreement for the evaluation of immunoreactivity was measured with a generalized Kappa (κ) test for the two observers according to the criteria of Landis and Koch [22]. The κ values for strength of agreement were interpreted as poor (< 0.00), slight (0.00–0.20), fair (0.21–0.40), moderate (0.41–0.60), substantial (0.61–0.80), and almost perfect (0.81–1.000).

The slides of surgically resected specimens were evaluated as described in our previous report [13]. Positive (≥ 30%) and negative (< 30%) MSLN staining results were scored as percentages of immunopositive cells in the tumor tissue sample.

### Detection of mismatch repair deficiency

We retrospectively evaluated the mismatch repair (MMR) protein status using immunohistochemical staining of MLH1 (Clone G168-15; BD Biosciences, San Jose, CA, USA) and MSH2 (FE11; Invitrogen, Carlsbad, CA, USA). Immunohistochemistry was performed as previously described [20, 23]. The normal colonic crypt epithelium adjoining the tumor served as the internal control. When expressed, both MLH1 and MSH2 proteins stain positively in the nuclei [24]. Cancers negative for MLH1 or MSH2 were considered to exhibit a DNA MMR deficiency. With the help of MLH1 and MSH2 protein analyses, Marcus *et al*. predicted that over 90% of MSI-H cases had an MMR gene defect and that all MSS cancers exhibited intact staining with both antibodies [25].

### Comparative study between immunohistochemical expression and RNA level of MSLN

Biopsy tissue samples from the 77 consecutive patients who underwent surgery for stage II/III CRC in 2016–2018 were subjected to comparative study between immunohistochemical expression and RNA level by RT-qPCR. Biopsy specimen was obtained endoscopically and was divided into two samples for performing immunohistochemistry and RT-PCR. One was formalin-fixed, and the immunohistochemical expression was examined as described above. The other one was snap-frozen in liquid nitrogen and stored at −80°C until assayed. The specimens were cut into serial 4-μm thick sections, mounted on silane-coated glass slides, and air-dried. Subsequently, the cancer cells were manually dissected by referring to a hematoxylin and eosin-stained section. Total cellular RNA was extracted using the ISOGEN reagent (Nippon Gene, Tokyo, Japan) according to the manufacturer’s protocol. First-strand cDNA was synthesized using the Prime Script RT-reagent kit (Takara Bio Inc., Otsu, Japan) as per the manufacturer’s instructions. RT-qPCR was performed as described elsewhere [13, 26].

### Statistical analysis

Correlations between MSLN expression scores and clinicopathological variables were calculated and tested for significance using χ^2^ tests or Fisher’s exact method, as applicable. The differences between continuous variables exhibiting a normal distribution were compared using unpaired *t*-tests. Disease-specific survival (DSS) was defined as the interval between surgery and death due to CRC recurrence. The Kaplan–Meier estimator was used to calculate the survival probabilities. Additionally, comparisons were made using the log-rank test. Time to recurrence (TTR) was defined as the time from the date of curative surgery to the time of recurrence, and for patients with no recurrent disease, the last time at which they were known to be recurrence-free was considered. Furthermore, survival probability after recurrence was defined as the interval between CRC recurrence and death caused by it. Covariates with trend-significant effects (*P* < 0.1) on univariate analysis were selected for multivariate analysis of the survival factors. Factors used for both analyses were limited to those that could be evaluated preoperatively. The significance of the association between clinical and pathological variables and postoperative survival was tested using Cox’s proportional hazards regression. Specifically, this test was used to determine both hazard ratios (HR) and 95% confidence intervals. All statistical analyses were performed using JMP Pro 13.1.0 software (SAS Institute, Cary, NC, USA). *P* values < 0.05 were considered as statistically significant.

## SUPPLEMENTARY MATERIALS



## Abbreviations

CI: confidence interval
DSS: disease-specific survival
TTR: time to recurrence
HR: hazard ratio
ROC: receiver operating characteristic
