# Supplementary material for: Significance of mesothelin expression in preoperative endoscopic biopsy specimens for colorectal cancer prognosis
Source: Oncotarget. 2020 Oct 27;11(43):3807–17. doi: 10.18632/oncotarget.27774 (PMC7597413; doi:10.18632/oncotarget.27774)
Supplement: Supplementary file 1 [file oncotarget-11-3807-s001.pdf]

Significance of mesothelin expression in preoperative endoscopic biopsy specimens for colorectal cancer prognosis

SUPPLEMENTARY MATERIALS

Supplementary Table 1: Relationship between mesothelin expression in biopsy specimens and the frontal margins of the tumors

		Frontal margins of the tumors	
		**MSLN-positive**	**MSLN-negative**
Biopsy specimens	MSLN-positive	32	29
	MSLN-negative	36	415

MSLN, mesothelin.
